# NaCT/SLC13A5 facilitates citrate import and metabolism under nutrient-limited conditions

**DOI:** 10.1016/j.celrep.2021.109701

**Published:** 2021-09-14

**Authors:** Avi Kumar, Thekla Cordes, Anna E. Thalacker-Mercer, Ana M. Pajor, Anne N. Murphy, Christian M. Metallo

**Affiliations:** 1 Department of Bioengineering, University of California, San Diego, La Jolla, CA 92093, USA; 2 Division of Nutritional Sciences, Cornell University, Ithaca, NY 14850, USA; 3 Department of Cell, Developmental and Integrative Biology, University of Alabama at Birmingham, Birmingham, AL 35294, USA; 4 Skaggs School of Pharmacy and Pharmaceutical Sciences, University of California, San Diego, La Jolla, CA 92093, USA; 5 Department of Pharmacology, University of California, San Diego, La Jolla, CA 92093, USA; 6 Moores Cancer Center, University of California, San Diego, La Jolla, CA 92037, USA; 7 Present address: Molecular and Cell Biology Laboratory, Salk Institute for Biological Studies, La Jolla, CA 92037, USA; 8 Lead contact

## Abstract

Citrate lies at a critical node of metabolism, linking tricarboxylic acid metabolism and lipogenesis via acetyl-coenzyme A. Recent studies have observed that deficiency of the sodium-dependent citrate transporter (NaCT), encoded by *SLC13A5*, dysregulates hepatic metabolism and drives pediatric epilepsy. To examine how NaCT contributes to citrate metabolism in cells relevant to the pathophysiology of these diseases, we apply ^13^C isotope tracing to *SLC13A5*-deficient hepatocellular carcinoma (HCC) cells and primary rat cortical neurons. Exogenous citrate appreciably contributes to intermediary metabolism only under hypoxic conditions. In the absence of glutamine, citrate supplementation increases *de novo* lipogenesis and growth of HCC cells. Knockout of *SLC13A5* in Huh7 cells compromises citrate uptake and catabolism. Citrate supplementation rescues Huh7 cell viability in response to glutamine deprivation or Zn^2+^ treatment, and NaCT deficiency mitigates these effects. Collectively, these findings demonstrate that NaCT-mediated citrate uptake is metabolically important under nutrient-limited conditions and may facilitate resistance to metal toxicity.

## In brief

Using isotope tracing, ^13^C MFA, and proliferation assays, Kumar et al. show that NaCT-mediated extracellular citrate is cytosolically catabolized, contributing to *de novo* lipogenesis (DNL) in HCC cells. Furthermore, extracellular citrate import by NaCT promotes growth in oxygen- and glutamine-limited conditions and protects against zinc-induced toxicity.

## Graphical abstract

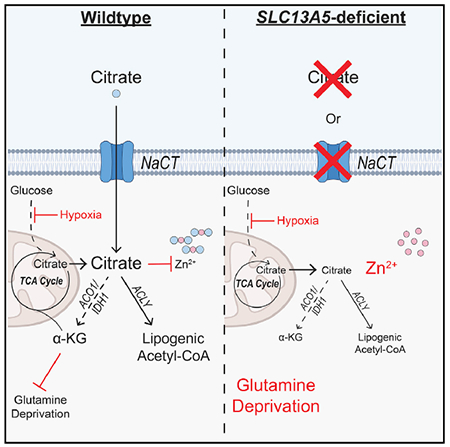

## INTRODUCTION

Citrate serves as a critical substrate for biosynthesis, acetylation, and the regeneration of NAD(P)H. Within mitochondria, citrate is synthesized by citrate synthase and metabolized in the tricarboxylic acid (TCA) cycle to support bioenergetics. Citrate is also exported to the cytosol via the mitochondrial citrate transporter (SLC25A1/CTP) and metabolized by ATP-citrate lyase (ACLY) to generate acetyl-coenzyme A (acetyl-CoA) for downstream metabolic processes, including lipid biosynthesis and acetylation (Wakil and Abu-Elheiga, 2009). Although mitochondrial production of citrate is the primary source for most cells, plasma concentrations are relatively high (Costello and Franklin, 2016). In addition, dysregulation of plasma citrate homeostasis has pathophysiological consequences including impaired blood clotting and bone disorders (Costello and Franklin, 2016; Zuckerman and Assimos, 2009). The functional importance of exogenous citrate transport by cells has therefore garnered increasing interest (Bhutia et al., 2017; Costello and Franklin, 1991, 2016).

A plasma membrane-specific variant of *SLC25A1*, pmCIC, allows for import and catabolism of extracellular citrate in prostate cancer cells (Mycielska et al., 2018). Furthermore, several members of the SLC13 sodium sulfate/carboxylate symporter gene family are capable of importing citrate into cells in a sodium-coupled mechanism (Pajor, 2006, 2014). Both *SLC13A2*, primarily expressed in the kidney and small intestine, and *SLC13A3*, widely expressed across tissues, are capable of importing citrate, although with significantly lower affinity than dicarboxylic TCA intermediates (Pajor, 2006, 2014). Liver and brain tissue expresses the sodium-dependent citrate transporter (NaCT; also known as mINDY), encoded by *SLC13A5*, which preferentially transports citrate across the plasma membrane (Inoue et al., 2002; Pajor, 1996).

*SLC13A5* function has been linked to hepatic glucose and fatty acid metabolism, and development of NaCT inhibitors has garnered therapeutic interest (Huard et al., 2015; Sauer et al., 2021). Deletion of *Slc13a5* is protective against high-fat diet-induced insulin resistance and attenuates hepatic gluconeogenesis and lipogenesis (Birkenfeld et al., 2011). Furthermore, pharmacological inhibition of NaCT reduced hepatic lipid accumulation in mice fed a high-fat diet (Huard et al., 2015). Additionally, hepatic *SLC13A5* expression correlated positively with both body and liver fat in a cohort of non-alcoholic fatty liver disease (NAFLD) patients (von Loeffelholz et al., 2017). Functional impacts of NaCT inhibition or knockdown have been proposed in cells, although findings are not necessarily dependent on the presence of extracellular citrate (Li et al., 2017; Phokrai et al., 2018; Poolsri et al., 2018). Citrate transport may also influence acetyl-CoA metabolism and ionic homeostasis in the nervous system, as loss-of-function mutations in *SLC13A5* have been linked to pediatric epilepsy, Kohlschütter-Tönz syndrome, and other brain disorders (Hardies et al., 2015; Klotz et al., 2016; Matricardi et al., 2020; Schossig et al., 2017; Thevenon et al., 2014). Notably, citrate levels were significantly increased in plasma and cerebrospinal fluid (CSF) in such epilepsy patients (Bainbridge et al., 2017). Mice deficient in this transporter accumulated citrate in CSF, while plasma abundances were not affected; moreover, *Slc13a5*^−/−^ mice exhibited an increased propensity for seizures as well as impaired neuronal function (Henke et al., 2020). While citrate’s role in chelating metal cations has been hypothesized to drive this pathophysiology (Bhutia et al., 2017; Glusker, 1980), the mechanism(s) through which *SLC13A5* deficiency drives pathogenesis in mammalian cells warrants further study.

Here, we have applied mass spectrometry and metabolic flux approaches to genetically engineered hepatocellular carcinoma (HCC) cell lines and primary cortical neurons to decipher the impact of extracellular citrate import on metabolism and cell viability. *SLC13A5* was expressed in both cell types, and exogenous citrate was imported and metabolized to fatty acids and TCA cycle intermediates. However, citrate only contributed appreciably to these pathways under hypoxic conditions where pyruvate dehydrogenase (PDH) flux is reduced. Under these conditions, citrate was predominantly catabolized in the cytosol to support acetyl-CoA generation. We also used CRISPR-Cas9 to generate *SLC13A5*-deficient HCC cell lines, which lacked the ability to transport and metabolize exogenous citrate. In addition, we observed that *SLC13A5* expression was required to increase the growth of hypoxic HCC cells under glutamine-deprived conditions. Finally, NaCT function was also necessary to allow for citrate-mediated protection against Zn^2+^ toxicity. Collectively, our study highlights the biological roles of NaCT and citrate metabolism in mammalian cells, emphasizing the importance of metabolic stress in observing significant phenotypes.

## RESULTS

### Extracellular citrate uptake is tissue specific

**Figure 1.**
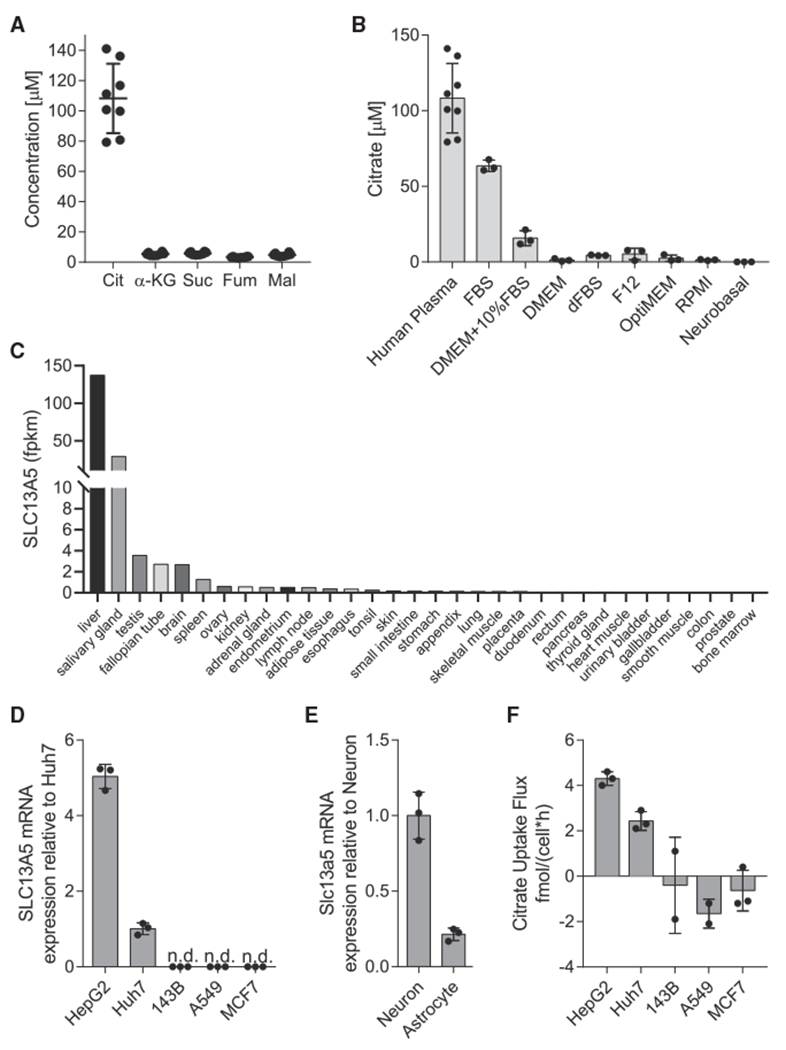
Extracellular citrate uptake is tissue specific (A) Plasma concentration of TCA intermediates in humans (n = 8). (B) Citrate concentration in cell culture medias (n = 3) and human plasma (n = 8) same as in (A). (C) *SLC13A5* mRNA expression in human tissues, from (Uhlén et al., 2015). (D) *SLC13A5* mRNA expression in cancer cells grown in normoxia relative to Huh7s (n = 3). (E) *Slc13a5* mRNA expression in primary rat neuron and astrocyte cells in normoxia relative to neurons (n = 3). (F) Net citrate uptake flux in cancer cell lines over 48 h (n = 3). Cit, citrate; α-KG, α-ketoglutarate; Suc, succinate; Fum, fumarate; Mal, malate. All data are presented as means ± SD. All data are representative of three sample replicates.

Recent findings have highlighted the importance of circulating TCA intermediates as metabolic substrates or regulators of tissue function (Mills et al., 2018). We quantified levels of TCA intermediates in human plasma and observed that the concentration of citrate was 108 ± 23 μM, while other TCA cycle intermediates were 10-fold less abundant (all <10 μM) (Figure 1A). Although citrate is not present in typical culture media such as DMEM, RPMI, or OptiMEM, we found that complete medium including 10% fetal bovine serum (FBS) contained 16 ± 5 μM citrate, significantly lower than that observed in human plasma (Figure 1B).

To identify cell types that might readily consume extracellular citrate, we first used a publicly available transcriptomics dataset from Uhlén et al. and compared *SLC13A5* expression across 32 human tissues (Uhlén et al., 2015). *SLC13A5* transcripts were highest in liver, brain, reproductive tissues, and salivary gland (Figure 1C). Next, we quantified the expression of *SLC13A5* in a panel of cells of different tissue origin. Consistent with published data (Uhlén et al., 2015), we found that the HCC cell lines HepG2 and Huh7 as well as primary cortical neurons express detectable *SLC13A5* mRNA, while little was detected in other cell lines (Figures 1D and 1E). We next cultured cells in DMEM supplemented with 500 μM extracellular citrate for 48 h and quantified uptake flux. Only the HCC cell lines exhibited net uptake of citrate, which correlated with *SLC13A5* transcription (Figure 1F).

### Citrate dilutes central carbon metabolism in HCC cells and neurons in hypoxia

**Figure 2.**
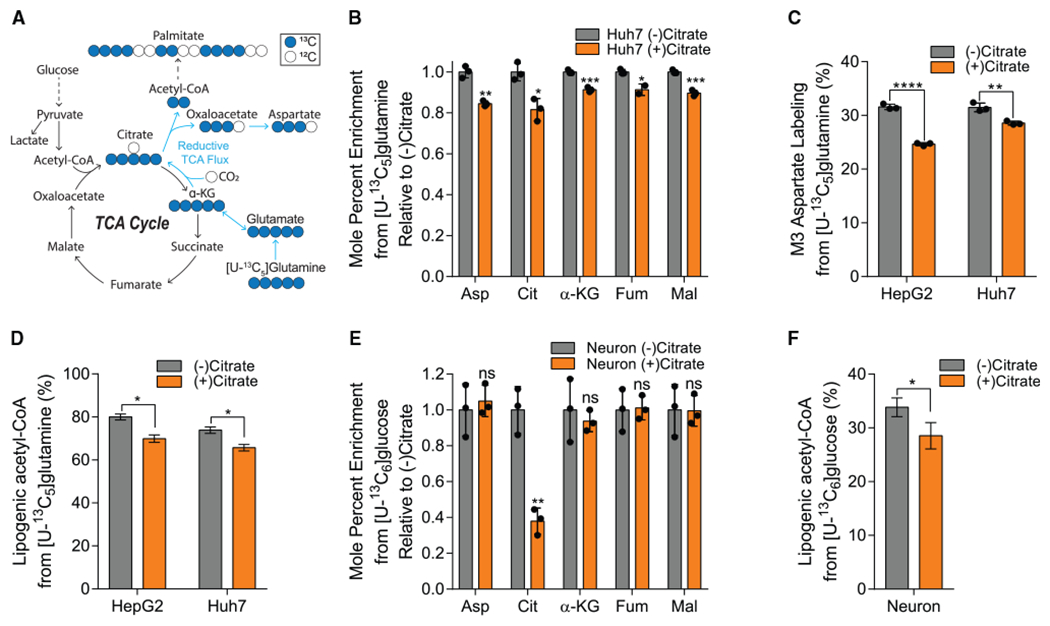
Citrate dilutes central carbon pathway labeling in hepatocellular carcinoma and neuronal cells in hypoxia (A) Atom transition map depicting catabolism of [U-^13^C_5_]glutamine. Closed circles indicate ^13^C carbon, and open circles indicate ^12^C carbon. (B) Mole percent enrichment of TCA intermediates from [U-^13^C_5_]glutamine in Huh7 cells grown in hypoxia ± 500 μM citrate for 48 h, relative to (−) citrate (n = 3). (C) Percent labeling of M3 aspartate from [U-^13^C_5_]glutamine in HepG2 and Huh7 cells grown in hypoxia ± 500 μM citrate for 48 h (n = 3). (D) Percentage of lipogenic acetyl-CoA contributed by [U-^13^C_5_]glutamine in HepG2 and Huh7 cells grown in hypoxia ± 500 μM citrate for 48 h (n = 3). (E) Mole percent enrichment of TCA intermediates from [U-^13^C_6_]glucose in primary rat neuron cells grown in 3% oxygen ± 500 μM citrate for 48 h, relative to (−) citrate (n = 3). (F) Percent of lipogenic acetyl-CoA contributed by [U-^13^C_6_]glucose in primary rat neuron cells grown in 3% oxygen ± 500 μM citrate for 48 h (n = 3). Asp, aspartate; Cit, citrate; α-KG, α-ketoglutarate; Fum, fumarate; Mal, malate. In (B), (C), and (E), data are plotted as mean ± SD. Statistical significance is relative to (−) citrate as determined by two-sided Student’s t test with *p < 0.05; **p < 0.01; ***p < 0.001; ****p < 0.0001. In (D) and (F), data are plotted as mean ± 95% confidence interval (CI). Asterisk (*) indicates statistical significance by non-overlapping CIs. Unless indicated, all data represent biological triplicates. Data shown are from one of at least two separate experiments. See also Figure S1.

The aforementioned results indicate that HCC cells import exogenous citrate. To quantify the contribution of citrate to central carbon metabolism in more detail, we cultured HepG2 and Huh7 cells in medium containing uniformly ^13^C-labeled ([U-^13^C_5_]) glutamine and quantified metabolite abundance and isotope enrichment (Figure 2A). Studies were performed under normoxic (21% oxygen) or hypoxic (1% oxygen) conditions for 48 h, as hypoxia is known to potently reduce citrate abundances due to decreased PDH flux (Metallo et al., 2011; Mullen et al., 2011; Wise et al., 2011). Indeed, TCA intermediate abundances were significantly decreased in hypoxia, with citrate being the most decreased of those measured (Figure S1A). As expected, labeling from [U-^13^C_5_]glutamine indicated that cells increased the contribution of reductive carboxylation to synthesis of aspartate and fatty acids as well as glutamine anaplerosis under hypoxia (Figures S1B–S1D). To indirectly determine whether exogenous citrate was metabolized in cells, we cultured Huh7 and HepG2 cells with and without 500 μM unlabeled (^12^C) citrate in medium containing [U-^13^C_5_]glutamine or [U-^13^C_6_]glucose, respectively. Citrate addition resulted in a dilution of glutamine’s contribution to TCA cycle anaplerosis (Figures 2B and S1E) and *de novo* lipogenesis through reductive carboxylation in HCC cell lines (Figures 2C and 2D). We performed similar studies using primary rat cortical neurons, although [U-^13^C_6_]glucose was used given the higher enrichment obtained in differentiated neurons (Divakaruni et al., 2017). Although labeling of the intracellular citrate pool was significantly diluted, glucose-derived TCA labeling was not impacted (Figure 2E). On the other hand, we observed a significant impact on the contribution of glucose to lipogenic acetyl-CoA using isotopomer spectral analysis (ISA) modeling (Figure 2F). Our findings suggest that extracellular citrate metabolically contributes as an anaplerotic and/or lipogenic substrate in low oxygen conditions and further highlight the need for some metabolic stress to observe a significant contribution.

**Figure 3.**
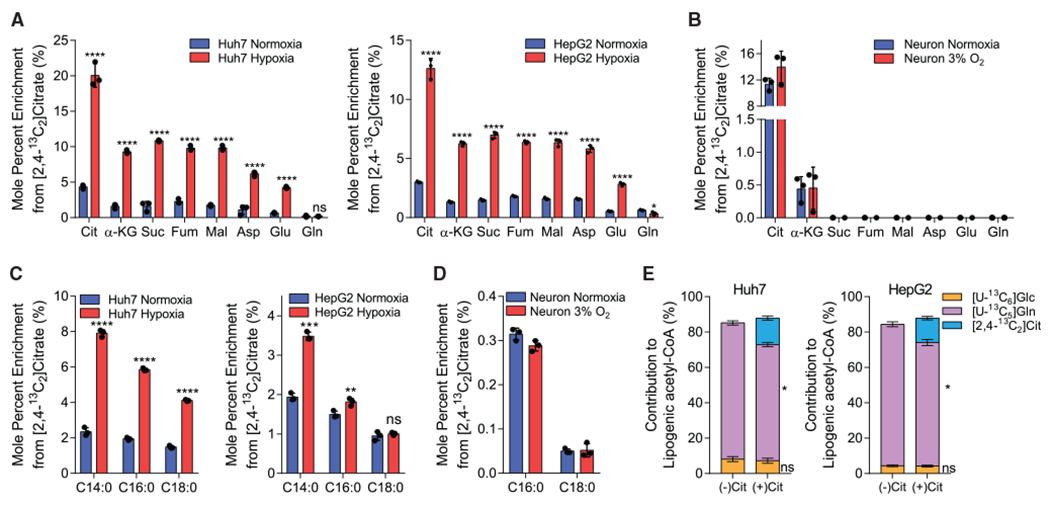
Exogenous citrate is metabolized for TCA anaplerosis and fatty acid synthesis (A) Mole percent enrichment of TCA intermediates from [2,4-^13^C_2_]citrate in Huh7 (left) and HepG2 (right) cells grown in normoxia or hypoxia for 48 h (n = 3). (B) Mole percent enrichment of TCA intermediates from [2,4-^13^C_2_]citrate in primary rat neuron cells grown in normoxia or 3% oxygen for 48 h (n = 3). (C) Mole percent enrichment of fatty acids from [2,4-^13^C_2_]citrate in Huh7 (left) and HepG2 (right) cells grown in normoxia or hypoxia for 48 h (n = 3). (D) Mole percent enrichment of fatty acids from [2,4-^13^C_2_]citrate in primary rat neuron cells grown in normoxia or 3% oxygen for 48 h (n = 3). (E) Percent of lipogenic acetyl-CoA contributed by [U-^13^C_6_]glucose, [U-^13^C_5_]glutamine, and [2,4-^13^C_2_]citrate, in the presence or absence of 500 μM unlabeled citrate in Huh7 (left) and HepG2 (right) cells grown in hypoxia for 48 h (n = 3). Cit, citrate; α-KG, α-ketoglutarate; Suc, succinate; Fum, fumarate; Mal, malate; Asp, aspartate; Glu, glutamate; Gln, glutamine; Glc, glucose. In (A)–(D), data are plotted as means ± SD. Statistical significance is relative to normoxia as determined by two-sided Student’s t test with *p < 0.05; **p < 0.01; ***p < 0.001; ****p < 0.0001. Unless indicated, all data represent biological triplicates. In (E), data are plotted as mean ± 95% CI. Asterisk (*) indicates statistical significance by non-overlapping CIs. Data shown are from one of at least two separate experiments. See also Figure S2.

To more directly quantify how extracellular citrate is metabolized in *SLC13A5*-expressing cells, we cultured the aforementioned cell types in growth medium supplemented with 500 μM [2,4-^13^C_2_]citrate. Cells were rinsed two times in NaCl prior to extraction to ensure accurate analysis of intracellular pools. In HepG2 and Huh7 cells, extracellular citrate contributed significantly to TCA labeling, with ~4% enrichment of citrate and ~1% enrichment of downstream intermediates in normoxic cells (Figure 3A). Under hypoxia, enrichment of intracellular citrate and other TCA intermediates was significantly elevated (Figure 3A). In Huh7 cells, net uptake of extracellular citrate was significantly lower than that of glucose or glutamine in both normoxia and hypoxia (Figure S2A). However, although citrate uptake was not significantly increased in hypoxia, the ratio of net citrate uptake to anaplerotic glutamine usage (glutamine uptake versus glutamate efflux) flux was increased in hypoxia (3.0) compared with normoxia (1.3) (Figure S2B), reflecting the increased dependence on exogenous citrate driven by reduced PDH flux in hypoxia. Intracellular citrate was also highly enriched by [2,4-^13^C_2_]citrate in neonatal rat cortical neurons, although detectable labeling was only observed on α-ketoglutarate (Figure 3B). Furthermore, no changes in enrichment were observed when comparing normoxic to 3% oxygen. These differences are likely due to the reduced anaplerotic/biosynthetic needs of post-mitotic neurons. Our results confirm that extracellular citrate is transported into these *SLC13A5*-expressing cell types. Importantly, we were unable to detect significant isotope enrichment on TCA intermediates in other cell types tested that lack *SLC13A5* expression, suggesting this transporter is important for citrate import and metabolism (Figure S2C).

Next, we examined whether extracellular citrate was metabolized to acetyl-CoA by quantifying enrichment of fatty acids in cells cultured with the isotope tracers noted above. We detected significant enrichment of palmitate and stearate from [2,4-^13^C_2_]citrate, demonstrating that HCC cell lines and neurons generate acetyl-CoA from exogenous citrate (Figures 3C and 3D). No enrichment of palmitate was observed in other cell types that lack *SLC13A5* expression (Figure S2D). We then compared the relative contributions of [U-^13^C_5_]glucose, [U-^13^C_5_]glutamine, and [2,4-^13^C_2_]citrate with the lipogenic acetyl-CoA fueling palmitate synthesis under hypoxia (Figure 3E). As expected, glutamine is the primary source of lipogenic carbon under these conditions. However, when citrate was present in the medium, HCC cells actively metabolized this substrate and the contribution of exogenous citrate to palmitate was greater than that of glucose, although glutamine remained the major lipogenic substrate in these hypoxic conditions. Notably, addition of extracellular citrate did not alter the rate of *de novo* lipogenesis (Figure S2E). Thus, exogenous citrate is taken up by *SLC13A5*-expressing cell types and is metabolized to fuel TCA cycle metabolism and lipogenesis, thereby diluting the corresponding glutamine contribution in low oxygen conditions. These findings are reminiscent of the usage of acetate under hypoxia in cells expressing cytosolic acetyl-CoA synthetase (ACSS2) (Kamphorst et al., 2014).

### Extracellular citrate is primarily catabolized in the cytosol

**Figure 4.**
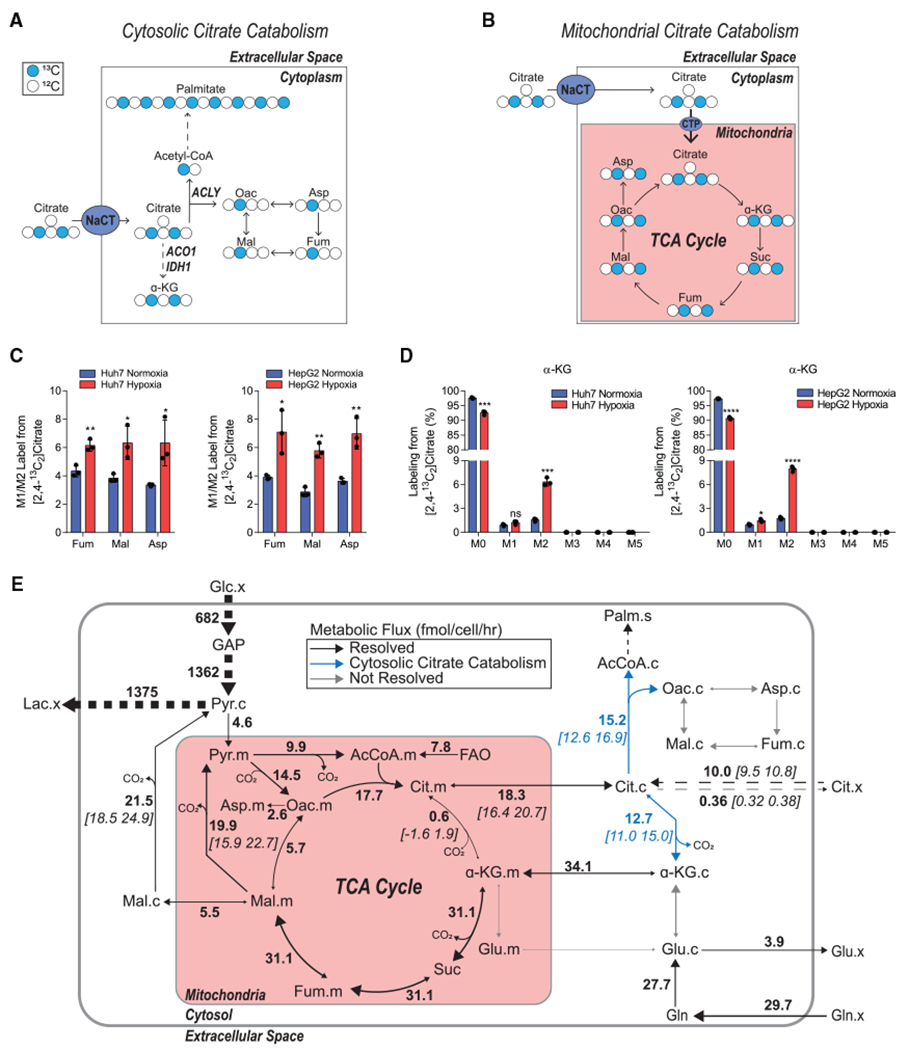
Extracellular citrate is primarily catabolized in the cytosol (A) Schematic of extracellular [2,4-^13^C_2_]citrate catabolism in the cytosol. Closed circles indicate ^13^C carbon, and open circles indicate ^12^C carbon. (B) Schematic of extracellular [2,4-^13^C_2_]citrate catabolism in the mitochondria. Closed circles indicate ^13^C carbon, and open circles indicate ^12^C carbon. (C) Ratio of the relative abundance of M1/M2 TCA intermediates from 500 μM [2,4-^13^C_2_]citrate in Huh7 (left) and HepG2 (right) cells grown in normoxia or hypoxia for 48 h (n = 3). (D) Mass isotopomer distribution of α-KG from 500 μM [2,4-^13^C_2_]citrate in Huh7 (left) and HepG2 (right) cells grown in hypoxia for 48 h (n = 3). (E) Schematic of central carbon metabolism with net fluxes and selected CIs estimated by ^13^C MFA for Huh7 cells grown in DMEM with 500 μM citrate in hypoxia for 48 h. Net flux values are listed adjacent to reaction arrows, with 95% CIs in square brackets. Dashed arrows represent grouped reactions. Some net or exchange fluxes for compartmentalized, parallel reactions were not resolvable. Cit, citrate; Oac, oxaloacetate; Fum, fumarate; Mal, malate; Asp, aspartate; α-KG, α-ketoglutarate; Glc, glucose; Gln, glutamine; Glu, glutamate; Lac, lactate; GAP, glyceraldehyde 3-phosphate; Palm, palmitate; FAO, fatty acid oxidation. In all graphs, data are plotted as means ± SD. Statistical significance is relative to normoxia as determined by two-sided Student’s t test with *p < 0.05; **p < 0.01; ***p < 0.001; ****p < 0.0001. Unless indicated, all data represent biological triplicates. Data shown are from one of at least two separate experiments.

The [2,4-^13^C_2_]citrate tracer enables more detailed insights into the compartment-specific catabolism of extracellular citrate when considering isotopolog distributions in more detail. In hypoxic, proliferating cancer cells, TCA intermediates are predominantly obtained from glutaminolysis (M4 labeling from [U-^13^C_5_] glutamine) or reductive carboxylation (M3 labeling from [U-^13^C_5_]glutamine), with TCA (re)cycling contributing to a lesser degree (Fan et al., 2013; Metallo et al., 2011). Therefore, TCA substrates in such cells are mostly present for one pass of the cycle. When [2,4-^13^C_2_]citrate is catabolized directly in the cytosol by ACLY, M1 acetyl-CoA and M1 oxaloacetate are formed, which further yielded M1 TCA intermediates via malate-aspartate shuttling and malic enzyme activity (Figure 4A). Alternatively, if citrate is imported into the mitochondria directly by SLC25A1, as has been shown to occur in anchorage-independent cultures (Jiang et al., 2016, 2017), [2,4-^13^C_2_]citrate will generate M2 isotopologues of TCA intermediates (Figure 4B). When culturing HCC cell lines in the presence of 500 μM [2,4-^13^C_2_]citrate for 48 h in either normoxia or hypoxia, we found that the M1 isotopolog of aspartate, malate, and fumarate was more abundant than the M2 isotopolog, and the ratio of M1/M2 labeling increased for these metabolites in hypoxic conditions (Figure 4C). On the other hand, relative abundance of M2 α-ketoglutarate was similar to or greater than M1 labeling, which indicates that a high exchange flux exists for the cytosolic aconitase (ACO1) and isocitrate dehydrogenase (IDH1) reactions (Figures 4A and 4D). To resolve these intracellular fluxes in a more unbiased manner that allows for multiple turns of the TCA cycle, we generated a ^13^C metabolic flux analysis (MFA) model encompassing glycolysis, compartmentalized TCA and amino acid metabolism, pyruvate cycling, *de novo* lipogenesis, and citrate import/exchange (Data S1) using the INCA software suite (Young, 2014). This model incorporated steady-state labeling of Huh7 cells grown in hypoxia with citrate across three independent experiments using either [U-^13^C_6_]glucose, [U-^13^C_5_]glutamine, or [2,4-^13^C_2_]citrate. We also included direct flux measurements of glucose, lactate, glutamine, glutamate, and citrate uptake and/or secretion. The model output confirmed that extracellular citrate (cit.x) contributes appreciably to lipogenesis and TCA metabolism and is significantly catabolized in the cytosol by ACLY and ACO1/IDH1 (Figure 4E). Importantly, the model estimated that reductive carboxylation exchange flux occurs primarily in the mitochondrial matrix via IDH2/ACO2 under these citrate-replete conditions, although cytosolic exchange cannot be entirely ruled out from this model (Tables S2 and S3). Glutamine-to-fatty acid flux was reconciled by high rates of pyruvate cycling and malic enzyme flux, consistent with the maintained activity of glutaminolysis that occurs in hypoxia (Fan et al., 2013; Vacanti et al., 2014). Collectively, these results suggest that exogenous citrate is primarily metabolized in the cytosol by ACLY and ACO1/IDH1 and predominantly enters TCA metabolism via α-ketoglutarate under the conditions tested (Figures 4C and 4D).

### NaCT supports extracellular citrate import and metabolism in HCC cells

**Figure 5.**
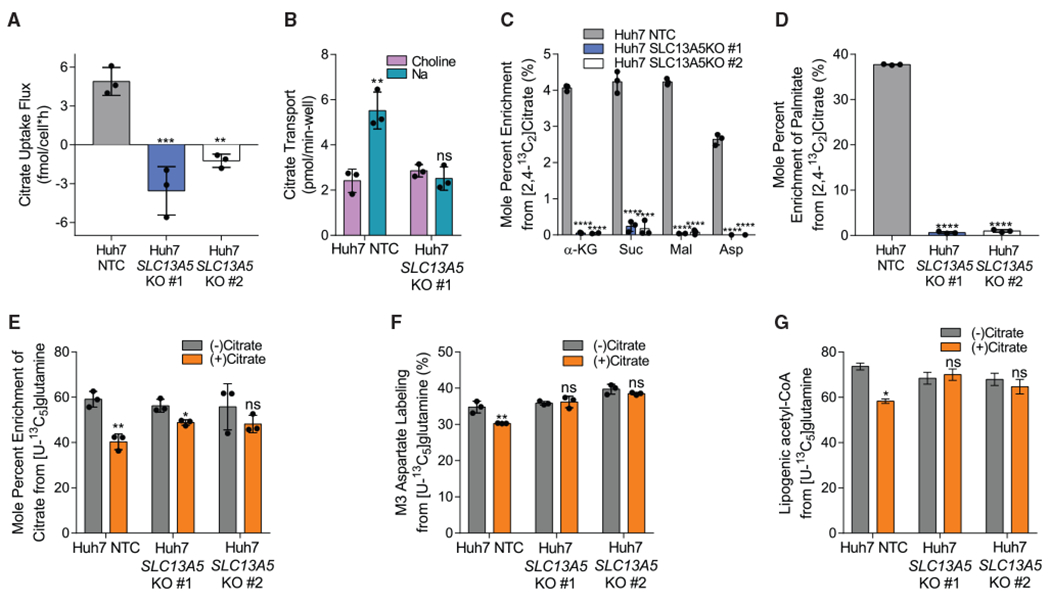
NaCT supports extracellular citrate import and metabolism in hepatocellular carcinoma cells (A) Citrate uptake flux in Huh7 NTC and *SLC13A5* KO cells grown in hypoxia for 48 h (n = 3). (B) Citrate transport in Huh7 NTC and *SLC13A5* KO #1 cells (n = 3). (C) Mole percent enrichment of TCA intermediates from [2,4-^13^C_2_]citrate in Huh7 NTC and *SLC13A5* KO cells grown in hypoxia for 48 h (n = 3). (D) Mole percent enrichment of palmitate from [2,4-^13^C_2_]citrate in Huh7 NTC and *SLC13A5* KO cells grown in hypoxia for 48 h (n = 3). (E) Mole percent enrichment of citrate from [U-^13^C_5_]glutamine in Huh7 NTC and *SLC13A5* KO cells ± 500 μM citrate grown in hypoxia for 48 h (n = 3). (F) Percent labeling of M3 aspartate from [U-^13^C_5_]glutamine in Huh7 NTC and *SLC13A5* KO cells ± 500 μM citrate grown in hypoxia for 48 h (n = 3). (G) Percentage of lipogenic acetyl-CoA contributed by [U-^13^C_5_]glutamine in Huh7 NTC and *SLC13A5* KO cells ± 500 μM citrate grown in hypoxia for 48 h (n = 3). α-KG, α-ketoglutarate; Suc, succinate; Mal, malate; Asp, aspartate. In all graphs data are plotted as means ± SD. Statistical significance is relative to NTC as determined by one-way ANOVA with Dunnett’s method for multiple comparisons (A, C, and D) or relative to (−) citrate as determined by two-sided Student’s t test (B, E, and F) with *p < 0.05; **p < 0.01; ***p < 0.001; ****p < 0.0001. In (G), data are plotted as mean ± 95% CI. Asterisk (*) indicates statistical significance by non-overlapping CIs. Unless indicated, all data represent biological triplicates. See also Figures S3 and S4. (C–G) Data shown are from one of at least two separate experiments.

To directly interrogate the function of NaCT with respect to citrate metabolism, we engineered *SLC13A5*-deficient knockout (KO) HepG2 and Huh7 cells using CRISPR-Cas9 and compared their metabolic phenotype with cells expressing a non-targeting control (NTC) single guide RNA (sgRNA). Two clones were tested for each KO cell line. *SLC13A5* KO clones were validated by sequencing the region of interest (Figure S3). In both cell lines, KO of *SLC13A5* resulted in reduced citrate uptake flux when the cells were cultured in growth medium supplemented with 500 μM exogenous citrate (Figures 5A and S4A), suggesting these cells lacked NaCT activity. To further verify the functional impact of NaCT deficiency, we quantified citrate transport using [1,5-^14^C] citrate (Pajor et al., 2016). Sodium-induced citrate uptake was significantly reduced in the *SLC13A5* KO cells (Figure 5B). Notably, NaCT deficiency induced no significant metabolic phenotype in cells grown in the absence of extracellular citrate, as fatty acid synthesis rates were unchanged and alterations to metabolite abundances were either moderate or inconsistent in the KO clones (Figures S4B and S4C). Additionally, although previous studies have found that *SLC13A5* knockdown alone induced a reduction in the expression of fatty acid synthesis gene expression in HCC cells (Li et al., 2017), we observed no impact on ACLY expression in HepG2 or Huh7 *SLC13A5* KO cells (Figure S4D). These data indicate that the citrate transporter induces a metabolic change that is selective to citrate uptake.

To elucidate how impaired NaCT activity influences citrate catabolism, we cultured each cell line for 48 h in the presence of 500 μM [2,4-^13^C_2_]citrate under hypoxic conditions. Huh7 *SLC13A5* KO cells had no measurable enrichment from [2,4-^13^C_2_]citrate on TCA cycle intermediates or fatty acids (Figures 5C and 5D). A reduction in enrichment was also observed with HepG2 *SLC13A5* KO cells (Figures S4E and S4F). Our data confirm that NaCT is the primary importer of extracellular citrate in the tested HCC cell lines. Next, we cultured NaCT-deficient cells in the presence or absence of exogenous ^12^C citrate in [U-^13^C_5_]glutamine tracer medium and quantified enrichment on TCA cycle intermediates and fatty acids compared with control cells. When unlabeled citrate was present in the media, we only observed dilution of [U-^13^C_5_]glutamine on TCA cycle intermediates in *SLC13A5*-expressing cells (Figures 5E and S4G). Furthermore, we observed no dilution in isotopologues downstream of reductive carboxylation (i.e., M3 aspartate) or glutamine-derived lipogenic acetyl-CoA with extracellular unlabeled citrate addition in the Huh7 *SLC13A5* KO cells (Figures 5F and 5G). Thus, our results indicate that NaCT is required for the import and metabolism of extracellular citrate, in HCC cells.

### NaCT facilitates growth under nutrient stress and resistance to Zn^2+^ toxicity

**Figure 6.**
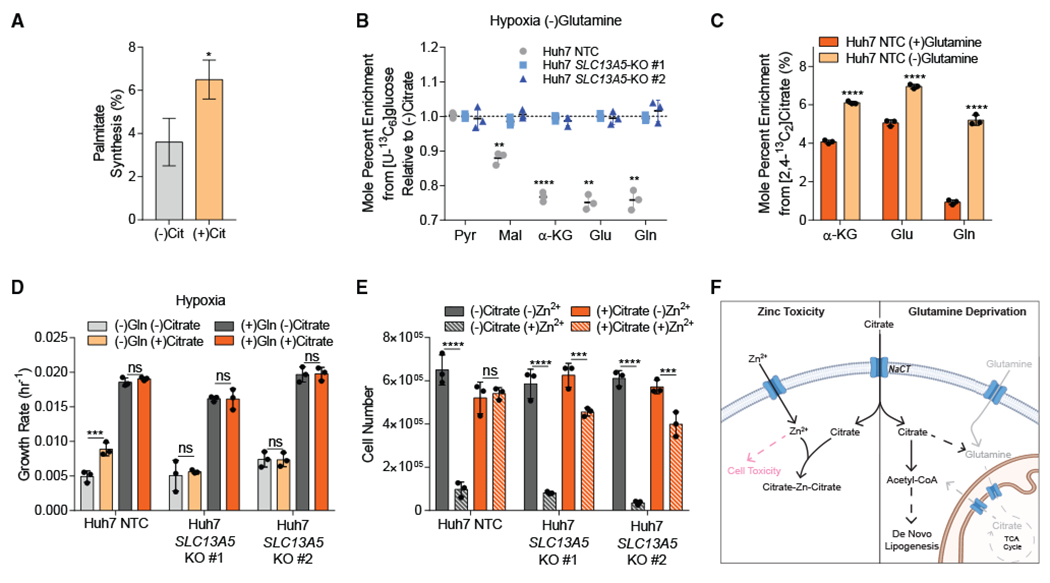
NaCT facilitates growth under nutrient stress and resistance to Zn^2+^ toxicity (A) Palmitate synthesis in Huh7 NTC cells grown in high glucose DMEM without glutamine ± 500 μM citrate in hypoxia for 48 h (n = 3). (B) Mole percent enrichment of metabolites from [U-^13^C_6_]glucose in Huh7 *SLC13A5* KO cells grown without glutamine in hypoxia ± 500 μM citrate for 48 h, relative to (−) citrate (n = 3). (C) Mole percent enrichment of TCA intermediates from [2,4-^13^C_2_]citrate in Huh7 NTC cells grown in hypoxia ± 4 mM glutamine for 48 h (n = 3). (D) Growth rates of Huh7 NTC and *SLC13A5*-KO cells grown in high glucose DMEM ± 4 mM glutamine ± 500 μM citrate in hypoxia for 4 days. (E) Cell numbers of Huh7 NTC and *SLC13A5*-KO cells grown in high glucose DMEM ± 5 mM citrate ± 300 μM ZnCl_2_ 24 h after Zn^2+^ treatment in hypoxia. (F) Schematic depicting extracellular citrate utilization during cellular stress. Gray indicates removed metabolites, and pink indicates cytotoxic. Created with Biorender.com. Cit, citrate; Pyr, pyruvate; Mal, malate; α-KG, α-ketoglutarate; Glu, glutamate; Gln, glutamine. In (A), data are plotted as mean ± 95% CI. Asterisk (*) indicates statistical significance by non-overlapping CIs. In (B)–(E), data are plotted as means ± SD. Statistical significance is determined by two-sided Student’s t test relative to (−) citrate (B), (+) glutamine (C), or determined by two-way ANOVA with Tukey’s method for multiple comparisons relative to (−) citrate (D) or (−) Zn^2+^ (E) conditions with *p < 0.05; **p < 0.01; ***p < 0.001; ****p < 0.0001. Unless indicated, all data represent biological triplicates. See also Figure S5. Data shown are from one of at least two separate experiments.

Next, we hypothesized that citrate import and catabolism could fulfill certain metabolic roles of glutamine, which serves as a key substrate for anaplerosis and acetyl-CoA synthesis under hypoxia. We cultured the aforementioned HCC cell lines in glutamine-deficient media containing [U-^13^C_6_]glucose supplemented with exogenous citrate. Notably, addition of citrate in the absence of glutamine significantly increased fatty acid synthesis in control cells (Figure 6A). Furthermore, citrate supplementation generally decreased enrichment of TCA intermediates and associated amino acids from [U-^13^C_6_]glucose, but increased the abundance of α-ketoglutarate in NTC cells (Figures 6B and S5A), no change was observed in NaCT-deficient cell lines. Next, we cultured *SLC13A5*-expressing Huh7 cells with [2,4-^13^C_2_]citrate and found that in the absence of glutamine, enrichment from ^13^C citrate on metabolites associated with glutamine synthesis increased significantly (Figure 6C). These data indicate that NaCT facilitates citrate-mediated anaplerosis under nutrient-limited conditions.

Since glutamine is critical for numerous biological processes, including anaplerosis and lipogenesis, we next tested whether NaCT-mediated citrate uptake impacted cell proliferation upon glutamine deprivation. We observed that exogenous citrate addition increased the growth rate of the Huh7 NTC cells by 80% in glutamine-depleted media under hypoxic conditions (Figure 6D). Notably, no growth alteration was observed with citrate addition in glutamine-rich media (Figure 6D) or normoxia (Figure S5B). Importantly, citrate supplementation did not increase proliferation of NaCT-deficient cell lines (Figures 6D and S5B). These data indicate that NaCT-mediated citrate transport supports metabolism under nutrient-limited conditions. We next tested whether NaCT-mediated citrate transport influences resistance to metal toxicity against ions such as zinc (Zn^2+^). Regions of the brain including the hippocampus and cerebral cortex are Zn^2+^ enriched (Assaf and Chung, 1984). Under pathological conditions including epilepsy, ischemia, and traumatic brain injury, cerebral Zn^2+^ levels increase and may cause neuronal cell death (Assaf and Chung, 1984; Frederickson et al., 1983; Weiss et al., 2000). Intracellular citrate has been suggested to be a protective chelator against Zn^2+^ in rat cortical neurons (Sul et al., 2016); however, the role of NaCT in this process remains unclear. Given the established link between pediatric epilepsy and *SLC13A5* mutations, we hypothesized that NaCT-mediated citrate uptake could generally facilitate protection against Zn^2+^ toxicity. Hepatocytes can also exhibit sensitivity to Zn^2+^ (Lemire et al., 2008; Steinebach and Wolterbeek, 1993), so we used the Huh7 NaCT-deficient cells described above to determine whether NaCT function mediates protection against Zn^2+^ toxicity. We cultured hypoxic Huh7 NTC and NaCT-deficient cells in the presence or absence of citrate and ZnCl_2_ for 24 h and quantified cell viability. While citrate supplementation protected Huh7 NTC against Zn^2+^ toxicity, cell lines lacking NaCT showed reduced viability (Figure 6E). Thus, our data highlight that extracellular citrate import via NaCT facilitates protection against nutrient deprivation as well as Zn^2+^ toxicity in human cells (Figure 6F).

## DISCUSSION

Cells take up diverse nutrients from the extracellular microenvironment, and each metabolite may serve a distinct purpose or function. Nutrient transport is selective and regulated, in part, through cell-specific expression of transporters such as NaCT, which is restricted to liver and neural tissue. Here, we investigated how NaCT influences citrate metabolism in HCC cell lines and primary rat cortical neurons. We observed that NaCT-mediated citrate import contributes to cytosolic acetyl-CoA pools in HCC cells and neurons under distinct conditions. Hypoxic HCC cells metabolized extracellular citrate to fatty acids and TCA intermediates in a NaCT-dependent manner. When glutamine (and other nutrients) became limited, citrate supported the growth and lipid synthesis of HCC cell lines expressing NaCT. Furthermore, citrate supplementation enhanced the viability of HCC cell lines exposed to Zn^2+^ toxicity, but this protective effect was mitigated in *SLC13A5* KO cells that lacked NaCT expression.

Many cancer cell types increase glutamine utilization to support the TCA cycle and fatty acid biosynthesis *in vitro* (Metallo et al., 2011; Mullen et al., 2011); however, reproducing this phenotype *in vivo* has generated mixed results (Davidson et al., 2016; Le et al., 2012; Son et al., 2013; Tardito et al., 2015). One potential explanation for this discrepancy is the difference in nutrient profile of cell culture media compared with *in vivo* microenvironments (Ackermann and Tardito, 2019; Rossiter et al., 2021; Vande Voorde et al., 2019). Plasma and interstitial fluid contain metabolites that impact cellular metabolism, but are routinely excluded from most cell culture medias. For instance, uric acid, a metabolite abundant in human plasma but absent in many cell culture medias, inhibited *de novo* pyrimidine synthesis in cancer cells *in vitro* (Cantor et al., 2017). Previous *in vitro* studies have observed that upregulated reductive glutamine catabolism supports fatty acid biosynthesis and defuses mitochondrial redox stress in hypoxia (Jiang et al., 2016; Metallo et al., 2011). In our study, we found that reductive carboxylation was elevated in hypoxia in HepG2 and Huh7 cells, but observed that extracellular citrate addition reduced flux through this pathway. Thus, uptake of extracellular citrate may provide an additional carbon source which is accessible to ACLY and downstream pathways, curtailing the need for other nutrients to support these processes. Similar observations of citrate import and metabolism were made in prostate cancer cells, which express pmCIC (Mycielska et al., 2018). On the other hand, we only observed appreciable citrate contributions to biosynthesis under selected conditions (hypoxia and glutamine restriction). These findings collectively suggest that citrate is not a major biosynthetic substrate, but serves as a resource to cells when they experience distinct stresses (e.g., ischemia, hypoxia, metal toxicity).

We also demonstrated that *SLC13A5*-encoded NaCT is the primary mechanism for citrate import in HCC cells. No major changes in metabolism were observed beyond acetyl-CoA and TCA metabolism, and phenotypic responses were dependent upon expression of functional NaCT as well as citrate supplementation. Importantly, some prior studies observed signaling and growth phenotypes in cells upon knockdown or inhibition of NaCT in the absence of extracellular citrate (Li et al., 2017; Phokrai et al., 2018; Poolsri et al., 2018), but our results indicate that citrate transport is a key function of the *SLC13A5* gene product. Indeed, we also found that citrate uptake by NaCT was protective against Zn^2+^ cytotoxicity in Huh7 cells. This finding mirrors those of previous studies that demonstrated that citrate or pyruvate administration were protective against Zn^2+^ cytotoxicity in neurons (Sul et al., 2016; Yoo et al., 2004). Loss-of-function mutations in *SLC13A5* have been associated with early-onset epilepsy, but the role of citrate metabolism in the pathophysiology of these patients is not well understood (Hardies et al., 2015; Klotz et al., 2016; Thevenon et al., 2014). Various mechanisms have been proposed for this phenotype, including the susceptibility of NaCT-deficient neurons to increased synaptic Zn^2+^ concentrations induced upon neuronal activation (Sul et al., 2016). Alternatively, others have hypothesized that improper NaCT function leads to increased synaptic citrate concentrations and excessive Zn^2+^ chelation, which impairs NMDA receptor function and drives neuronal dysfunction (Bhutia et al., 2017). Notably, we performed our study in HCC cells using supraphysiological concentrations of citrate and Zn^2+^ that might only occur transiently in the body. Furthermore, application of relatively severe metabolic stress (i.e., 1% oxygen and glutamine deprivation) to limit endogenous citrate was required to observe a growth phenotype in HCC cells. These findings suggest that biosynthetic defects may not be as relevant to the neurological phenotype of NaCT-deficient patients. As such, our results should motivate further investigation into the impact of *SLC13A5* mutations on homeostasis of potentially toxic metal cations such as Zn^2+^ and its relation to epilepsy. Ideally, such studies would be performed in a more physiological model system such as NaCT KO in brain organoids or animals, which recapitulate the physiology of citrate better than 2D cell culture. Collectively, our results demonstrate that NaCT mediates citrate transport and metabolism under conditions of metabolic stress. Our approach also highlights the utility of coordinated studies that involve targeting of disease-related metabolic genes in relevant cell types and deep metabolic profiling using flux analysis.

## STAR★METHODS

### RESOURCE AVAILABILITY

#### Lead contact

Further information and requests for resources and reagents should be directed to and will be fulfilled by the Lead Contact, Christian M. Metallo (cmetallo@ucsd.edu).

#### Materials availability

This study did not generate new unique reagents.

#### Data and code availability

- All data reported in this paper will be shared by the lead contact upon request.
- All original code is available in this paper’s supplemental information.
- Any additional information required to reanalyze the data reported in this paper is available from the lead contact upon request.

### EXPERIMENTAL MODEL AND SUBJECT DETAILS

#### Human Samples

Plasma samples from healthy fasted adults (29-42 years, 5 male, 3 female) were obtained from a clinical cohort recruited from Tompkins County, New York area as described previously (Gheller et al., 2021). Participants were excluded if they had a history of negative or allergic reactions to local anesthetic, used immunosuppressive medications, were prescribed anti-coagulation therapy, were pregnant, had a musculoskeletal disorder, suffered from alcoholism (> 11 drinks per week for women and > 14 drinks per week for men) or other drug addictions or were acutely ill at the time of participation (Gheller et al., 2019; Riddle et al., 2018). The Cornell University Institutional Review Board approved the protocol and all the subjects provided written informed consent in accordance with the Declaration of Helsinki.

#### Cell Lines

HepG2, Huh7, A549, MCF7, and 143B cells were obtained from ATCC and were incubated at 37C with 5% CO_2_ and cultured using Dulbecco’s Modified Eagle Media (DMEM) with 10% Fetal Bovine Serum and 1% Penicillin-Streptomycin. Cells tested negative for mycoplasma contamination. For hypoxia experiments, cells were maintained in a humidified glove box (Coy) at 5% CO_2_ and either 1% O_2_ (cancer cell lines) or 3% O_2_ (primary cortical neurons). Primary cortical neurons and astrocytes were isolated and cultured in Neurobasal medium (neuron) or DMEM medium supplemented with 10 mM glucose, 0 mM glutamine, and 10% FBS (astrocytes) as described in detail by Cordes et al. (2020). All media were adjusted to pH = 7.3.

### METHOD DETAILS

#### Cell Proliferation and ^13^C Tracing

Proliferation studies were performed on 12 well plates with an initial cell number of 50,000/well for Huh7s and 100,000/well for HepG2s. Cells were plated in growth media and allowed to adhere for 24 hours before changing to the specified growth media. Cell counts were performed at days 0 and 4 using a hemocytometer.

For Zn^2+^ tolerance studies, cells were plated at 50,000/well and allowed to adhere for 24 hours. They were then placed in the hypoxia chamber for 24 hours before swapping to ± 5 mM citrate media. After changing media, cells were allowed to acclimate for 24 hours before 300 μM ZnCl_2_ in water was spiked into the designated wells. Cells were counted 24 hours after Zn^2+^ treatment using a hemocytometer.

^13^C isotope tracing media was formulated using glucose and glutamine free DMEM 5030 supplemented with either 20 mM [U-^13^C_6_] glucose, 4 mM [U-^13^C_5_]glutamine, or 500 μM [2,4-^13^C_2_]citrate (Cambridge Isotopes) and 10% dialyzed FBS. All studies were performed with a final concentration of 20 mM glucose and 4 mM glutamine. Cultured cells were washed with 1 mL PBS prior to applying tracing media for 24-72 hours as indicated in figure legends. For tracing experiments performed in hypoxia, cancer cells were acclimated to the hypoxia chamber in basal media for 24 hours prior to applying pre-hypoxified tracing media. Mole percent enrichment (MPE) of isotopes were calculated as the percent of all atoms within the metabolite pool that are labeled, using Escher-Trace (Kumar et al., 2020):

$$\frac{\sum_{i=0}^{n}i\cdot M_{i}}{n}$$

where *n* is the number of carbon atoms in the metabolite and *M_i_* is the relative abundance of the *i*th mass isotopomer. The MPE of [2,4-^13^C_2_]citrate is 33%, while MPE for [U-^13^C_6_]glucose and [U-^13^C_5_]glutamine is 100%.

#### Isotopomer Spectral Analysis (ISA)

Isotopomer spectral analysis (ISA) was performed to estimate the percent of newly synthesized palmitate as well as the contribution of a tracer of interest to the lipogenic acetyl-CoA pool (Cordes and Metallo, 2019; Young, 2014). Parameters for contribution of ^13^C tracers to lipogenic acetyl-CoA (D value) and percentage of newly synthesized fatty acid (g(t) value) and their 95% confidence intervals are then calculated using a best-fit model from INCA MFA software. Experimental fatty acid labeling from [U-^13^C_6_]glucose, [U-^13^C_5_]glutamine, or [2,4-^13^C_2_]citrate after 48 hour trace was compared to simulated labeling using a reaction network where C16:0 is condensation of 8 AcCoA. ISA data plotted as mean ± 95% CI. * indicates statistical significance by non-overlapping confidence intervals.

#### CRISPR/Cas9 engineered knockout cell lines

*SLC13A5* knockout clones were generated using the strategy described previously (Ran et al., 2013). Briefly, a guide RNA (gRNA) was designed to target the human *SLC13A5* gene using the CRISPOR webtool (gRNA sequence: AGGCACAATGAATAGCAGGG) (Concordet and Haeussler, 2018). The gRNA duplex was cloned into lentiCRISPRv2 (Addgene #52961) (Sanjana et al., 2014). HepG2 and Huh7 cells were transfected with the *SLC13A5* specific gRNA to generate pooled knockouts. After puromycin selection, single-cell clones were isolated by diluting the pooled knockout lines at 1 cell/100 μL and plating 100 μL into each well of a 96 well plate. Clones were maintained by exchanging media every 3-5 days. HepG2 clones were cultured in 50% conditioned media to enhance proliferation at early stages of cloning. Conditioned media was generated by collecting the spent media that had been culturing HepG2s after 2 days, spinning down at 300*g* for 5 min, and filtering using a 0.2 micron filter to clear cellular debris. This media was diluted 1:1 with DMEM with 10% FBS 1% penstrep to generate the 50% conditioned media. Clones were validated via Sanger sequencing. Sequences were aligned using the NCBI BLASTN suite (Agarwala et al., 2018).

#### Lentivirus Production

One 10cm dish of HEK293FT cells at 60% confluency were transfected with 1.3 μg VSV.G/pMD2.G, 5.3 μg lenti-gag/pol/pCMVR8.2, and 4 μg of the gRNA duplexed lentiCRISPRv2 using 16 μL Lipofectamine 3000 diluted in 0.66 mL of OPTI-MEM. Medium containing viral particles was harvested 48 and 72 hours after transfection, then concentrated by Centricon Plus-20 100,000 NMWL centrifugal ultrafilters, divided into aliquots and frozen at −80°C.

#### Determination of Extracellular Fluxes

Metabolic fluxes for citrate were calculated by collecting media at time 0 and spent media after 48 hours. Spent media was centrifuged at 300 g for 5 min, to remove cell debris. Cell counts were performed at time 0 and after 48 hours as well. To calculate citrate uptake fluxes, citrate was quantified by GC-MS via standard curve. To calculate glucose, lactate, glutamine, and glutamate uptake fluxes media metabolites were quantified using a Yellow Springs Instruments (YSI) Biochemistry Analyzer 2950.

#### Metabolite Extraction and GC-MS Analysis

At the conclusion of the tracer experiment, media was aspirated. Then, cells were rinsed twice with 0.9% saline solution and lysed with 250 μL ice-cold methanol. After 1 minute, 100 μL water containing 1 μg/ml norvaline was added to each sample and vortexed for one minute. 250 μL chloroform was added to each sample, and all were vortexed again for 1 minute. After centrifugation at 21,130 g for 10 minutes at 4°C, 250 μL of the upper aqueous layer was collected and evaporated under vacuum at 4°C. The lower organic layer was evaporated under air at room temperature.

Plasma metabolites were extracted and quantified as follows. For metabolite extraction, 10 μL of each plasma sample was utilized. First, 90 μL of a 9:1 methanol:water mix was added to each sample and the samples were vortexed for 1 minute. After centrifugation at 16,000 g for 10 minutes, 90 μL of supernatant was collected, evaporated under vacuum at −4°C and analyzed using GC/MS. Metabolite levels of TCA intermediates were quantified using external standard curves.

Dried polar and nonpolar metabolites were processed for gas chromatography (GC) mass spectrometry (MS) as described previously in Cordes and Metallo (2019). Briefly, polar metabolites were derivatized using a Gerstel MultiPurpose Sampler (MPS 2XL). Methoxime-tBDMS derivatives were formed by addition of 15 μL 2% (w/v) methoxylamine hydrochloride (MP Biomedicals, Solon, OH) in pyridine and incubated at 45°C for 60 minutes. Samples were then silylated by addition of 15 μL of N-tert-butyldimethylsily-N-methyltrifluoroacetamide (MTBSTFA) with 1% tert-butyldimethylchlorosilane (tBDMS) (Regis Technologies, Morton Grove, IL) and incubated at 45°C for 30 minutes. Nonpolar metabolites were saponified and transesterified to fatty acid methyl esters (FAMEs) by adding 500 μL of 2% H_2_SO_4_ in methanol to the dried nonpolar layer and heating at 50°C for 1 hour. FAMEs were then extracted by adding 100 μL of a saturated salt solution and 500 μL hexane and vortexing for 1 minute. The hexane layer was removed, evaporated, and resuspended with 60μL hexane for injection.

Derivatized polar samples were injected into a GC-MS using a DB-35MSUI column (30 m x 0.25mm i.d. x 0.25 μm, Agilent J&W Scientific, Santa Clara, CA) installed in an Agilent 7890B GC system integrated with an Agilent 5977a MS. Samples were injected at a GC oven temperature of 100°C which was held for 1 minute before ramping to 255°C at 3.5°C/min then to 320°C at 15°C/min and held for 3 minutes. Electron impact ionization was performed with the MS scanning over the range of 100-650 m/z for polar metabolites.

Derivatized nonpolar samples were injected into a GC-MS using a Fame Select column (100 m x 0.25mm i.d. x 0.25 μm, Agilent J&W Scientific, Santa Clara, CA) installed in an Agilent 7890A GC system integrated with an Agilent 5977A MS. Samples were injected at a GC oven temperature of 80°C which was held for 1 minute before ramping to 170°C at 20°C/min then to 188°C at 1°C/min then to 250°C at 20°C/min and held for 10 minutes. Electron impact ionization was performed with the MS scanning over the range of 54-400 m/z for nonpolar metabolites.

Metabolite levels and mass isotopomer distributions were analyzed with an in-house MATLAB script which integrated the metabolite fragment ions and corrected for natural isotope abundances.

#### ^13^C Metabolic Flux Analysis

^13^C Metabolic Flux Analysis (MFA) was performed using INCA (Young, 2014). The model was comprised of the chemical reactions and atom transitions of central carbon metabolism, as well as extracellular fluxes of glucose, lactate, glutamine, glutamate, and citrate and the isotopic labeling distributions of intracellular metabolites from Huh7 cells traced with DMEM and 500μM citrate containing [U-^13^C_6_]glucose, [U-^13^C_5_]glutamine, or [2,4-^13^C_2_]citrate under hypoxia.

The ^13^C MFA was performed with the following assumptions:

1. Cellular metabolism and isotopic labeling were assumed to be at steady state.
2. Cells were assumed to grow exponentially.
3. Labeled CO2 formed from decarboxylation reactions is dilute and assumed to never reincorporate in carboxylation reactions.
4. Separate mitochondrial and cytosolic pools of aspartate, oxaloacetate, malate, fumarate, α-KG, citrate, glutamate, pyruvate, and acetyl-CoA were modeled with exchange fluxes for malate, aspartate, glutamate, α-KG, and citrate.
5. The gross, per cell biomass requirements of proliferating Huh7 hepatoma cells were similar to those reported previously for mammalian cells (Grassian et al., 2014).

Additional information regarding the estimated fluxes generated by the model, as well as the data incorporated into the model can be found in the supplementary material (Tables S2 and S3; Data S1).

#### RNA isolation and quantitative RT-PCR

Total RNA was purified from cultured cells using Trizol Reagent (Life Technologies) per manufacturer’s instructions. First-strand cDNA was synthesized from 1 μg of total RNA using iScript Reverse Transcription Supermix for RT-PCR (Bio-Rad Laboratories) according to the manufacturer’s instructions. Individual 20 μL SYBR Green real-time PCR reactions consisted of 1 μL of diluted cDNA, 10 μL of SYBR Green Supermix (Bio-Rad), and 1 μL of each 5 μM forward and reverse primers. For standardization of quantification, 18S was amplified simultaneously. The PCR was carried out on 96-well plates on a CFX Connect Real time System (Bio-Rad), using a three-stage program provided by the manufacturer: 95°C for 3 min, 40 cycles of 95°C for 10 s and 60°C for 30 s. Gene-specific primers are tabulated in Table S1.

#### ^14^C citrate uptake assay

Uptake of ^14^C citrate was quantified as described previously (Pajor and Sun, 2010). Briefly, cells were seeded at 3 × 10^5^ cells/well on 6 well collagen treated plates. After 24 hours, cells were acclimated to serum free media for 24 hours. To carry out the assay, each well was washed twice with choline buffer (mM: 140 Choline, 2 KCl, 1 MgCl_2_, 1 CaCl_2_, 10 HEPES, pH adjusted to 7.4 with 1M Tris), then incubated with 1 mL sodium buffer (mM: 140 NaCl, 2 KCl, 1 MgCl_2_, 1 CaCl_2_, 10 HEPES, pH adjusted to 7.4 with 1M Tris) or choline buffer containing 100 μM [^14^C]-citrate for 30 minutes at 37°C with rocking. The uptake assays were stopped, and the cells were washed four times with 2.5 mL choline buffer. Cells were dissolved in 0.4 ml/well 1% SDS, transferred to scintillation vials with Econoscint Scintillation cocktail and then the radioactivity in the plates was counted using a scintillation counter.

### QUANTIFICATION AND STATISTICAL ANALYSIS

Statistical analyses were performed using Graphpad PRISM software. Unless indicated, all results shown as mean ± SD of cellular triplicates obtained from one representative experiment as specified in each figure legend. P values were calculated using a Student’s two-tailed t test, One-way ANOVA w/ Dunnet’s method for multiple comparisons, or Two-way ANOVA w/ Tukey’s method for multiple comparisons; *, P value < 0.05; **, P value < 0.01; ***, P value < 0.001, ****, P value < 0.0001. Unless indicated, all normalization and statistical tests compared to NTC cells, normoxia, or (−)citrate conditions.

## Supplementary Material

Supplementary figures and tables

Table S4

Data S1

## Figures and Tables

**Table:** KEY RESOURCES TABLE

REAGENT or RESOURCE	SOURCE	IDENTIFIER
Bacterial and virus strains		
One Shot Stbl3 E. Coli	ThermoFisher Scientific	Cat#C7373
Library Efficiency DH5α Competent Cells	ThermoFisher Scientific	Cat#18263012
Biological samples		
Healthy human plasma	Thalacker-Mercer Lab; Gheller et al., 2021	N/A
Chemicals, peptides, and recombinant proteins		
[U-^13^C_6_] Glucose	Cambridge Isotope Labs	Cat#CLM-1396
[U-^13^C_5_] Glutamine	Cambridge Isotope Labs	Cat#CLM-1822
[2,4-^13^C_2_] Citrate	Cambridge Isotope Labs	Cat#CLM-148
[1,5-^14^C_2_] Citrate	Morovak Inc.	N/A
Zinc Chloride	Sigma-Aldrich	CAS# 7646-85-7
Sodium Citrate	Sigma-Aldrich	CAS# 6132-04-3
Methoxylamine Hydrochloride	MP Biomedicals	Cat#02155405
MTBSTFA + 1% TBDMSCl	Regis Technologies	Cat#1-270143-200
BsmBI	New England Biolabs	Cat#R0580L
Critical commercial assays		
Lipofectamine 3000	ThermoFisher Scientific	Cat#L3000008
KAPA HiFi HotStart Ready Mix	Kapa Biosystems	Cat#KK2602
iTaq Universal SYBR Green	Biorad	Cat#1725121
iScript Reverse Transcription Supermix	Biorad	Cat#1708840
RNeasy Mini Kit	QIAGEN	Cat#74104
Zero Blunt TOPO PCR Cloning Kit	ThermoFisher Scientific	Cat#450245
prepGEM gold buffer	Zygem	N/A
Experimental models: Cell lines		
HepG2	ATCC	Cat#HB-8065
Huh7	Provided by M. Hermann, MIT, Cambridge, MA, USA	N/A
A549	ATCC	Cat#CCL-185
143B	ATCC	Cat#CRL-8303
MCF7	ATCC	Cat#HTB-22
HEK293FT	ThermoFisher Scientific	Cat#R70007
Primary rat cortical neurons	This paper	N/A
Primary rat astrocytes	This paper	N/A
Oligonucleotides		
See Table S1	N/A	N/A
Recombinant DNA		
LentiCRISPR v2 plasmid	Addgene	Cat#52961
pCMVR8.2	Addgene	Cat#12263
pMD2.G	Addgene	Cat#12259
Software and algorithms		
MATLAB 2014b and 2016b	MathWorks	https://www.mathworks.com/products/matlab.html
Escher-Trace	Kumar et al., 2020	https://escher-trace.github.io/
GraphPad Prism 7.00	GraphPad	https://www.graphpad.com/scientific-software/prism/
INCA 1.5	Young, 2014	https://mfa.vueinnovations.com/
CRISPOR	Concordet and Haeussler, 2018	http://crispor.tefor.net/
NCBI BLAST	Agarwala et al., 2018	https://blast.ncbi.nlm.nih.gov/Blast.cgi

## Highlights

- HCC cells utilize NaCT-mediated citrate uptake for lipogenesis
- Citrate import supports lipogenesis and anaplerosis upon glutamine deprivation
- NaCT-mediated citrate uptake facilitates protection against zinc toxicity
