# Supplementary material for: NaCT/SLC13A5 facilitates citrate import and metabolism under nutrient-limited conditions
Source: Cell Rep. Author manuscript; Available in PMC 2021 Oct 8. (PMC8500708; doi:10.1016/j.celrep.2021.109701)
Supplement: Supplementary figures and tables [file NIHMS1742219-supplement-Supplementary_figures_and_tables.pdf]

Cell Reports, Volume *36*

Supplemental information

NaCT/SLC13A5 facilitates citrate import and metabolism under nutrient-limited conditions

Avi Kumar, Thekla Cordes, Anna E. Thalacker-Mercer, Ana M. Pajor, Anne N. Murphy, and Christian M. Metallo

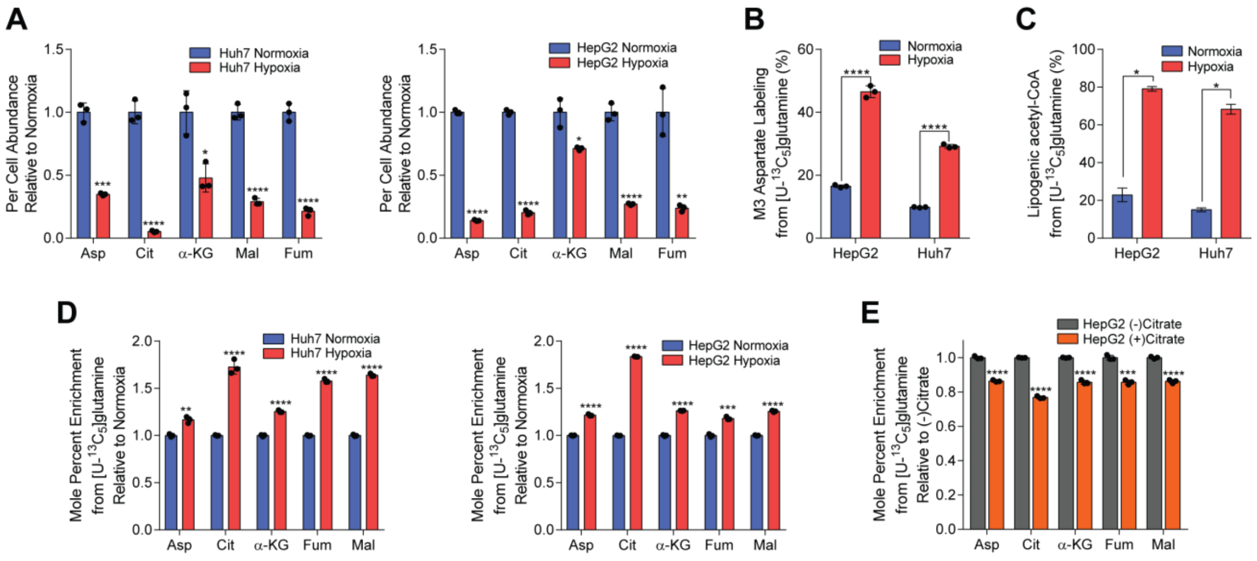

Supplementary Figure 1. Citrate dilutes central carbon pathway labeling in hepatocellular carcinoma and neuronal cells in hypoxia. Related to Figure 2.

(A) Per cell abundances of TCA intermediates in Huh7 (left) and HepG2 (right) cells grown in normoxia or hypoxia for 48 hours, relative to normoxia (n=3).

(B) Percent labeling of M3 aspartate from [U-$^{13}C_5$]glutamine in HepG2 and Huh7 cells grown in normoxia or hypoxia for 48 hours (n=3).

(C) Percent of lipogenic acetyl-CoA contributed by [U-$^{13}C_5$]glutamine in HepG2 and Huh7 cells grown in normoxia or hypoxia for 48 hours (n=3).

(D) Mole percent enrichment of TCA intermediates from [U-$^{13}C_5$]glutamine in Huh7 (left) and HepG2 (right) cells grown in normoxia or hypoxia for 48 hours, relative to normoxia (n=3).

(E) Mole percent enrichment of TCA intermediates from [U-$^{13}C_5$]glutamine in HepG2 cells grown in hypoxia +/- 500 µM citrate for 48 hours, relative to (-) citrate (n=3).

Asp, aspartate; Cit, citrate; α-KG, α-ketoglutarate; Fum, fumarate; Mal, malate. In (A,B,D,E) data are plotted as mean ± SD. Statistical significance is relative to normoxia (A,B,D) or (-) citrate (E) as determined by two-sided Student's t-test with *, P value < 0.05; **, P value < 0.01; ***, P value < 0.001, ****, P value < 0.0001. In (C) data are plotted as mean ± 95% confidence interval (CI). Statistical significance by non-overlapping confidence intervals, *. Unless indicated, all data represent biological triplicates. Data shown are from one of at least two separate experiments.

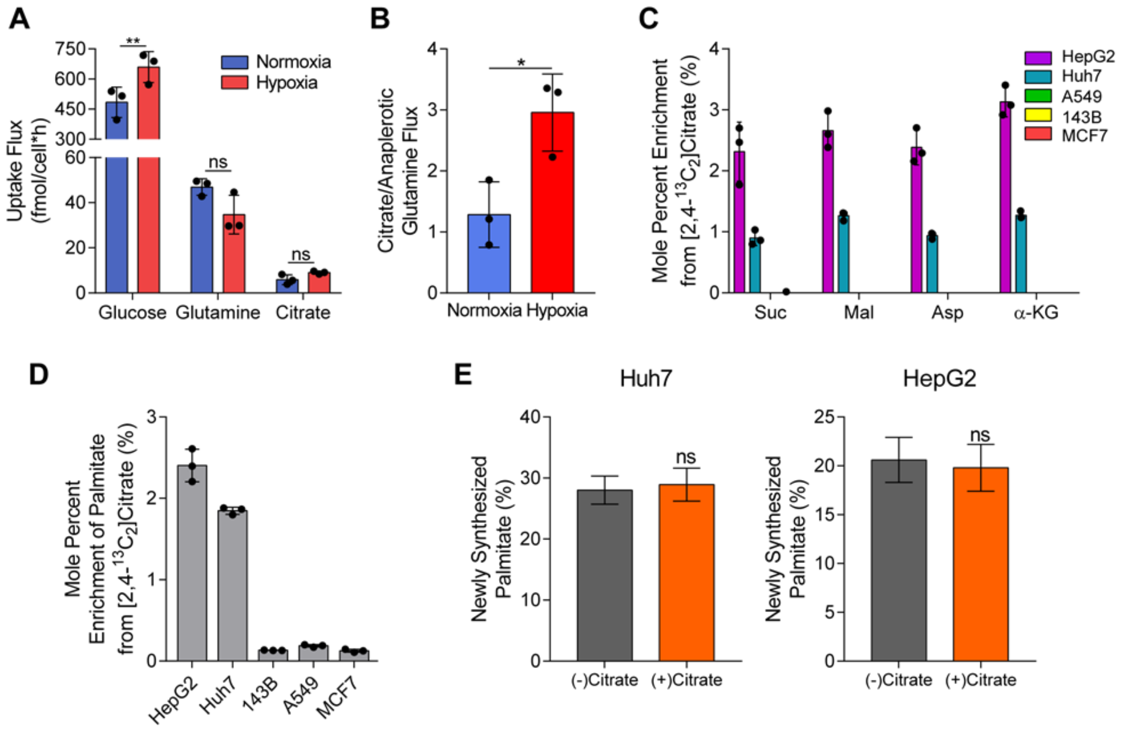

Supplemental Figure 2. Exogenous citrate is metabolized for TCA anaplerosis and fatty acid synthesis. Related to Figure 3.

(A) Glucose, glutamine, and citrate uptake flux over 48 hours in Huh7 cells grown in normoxia or hypoxia (n=3).

(B) Ratio of net citrate to anaplerotic glutamine flux over 48 hours in Huh7 cells grown in normoxia or hypoxia. Anaplerotic flux of glutamine calculated by subtracting media glutamate efflux from glutamine uptake (n=3).

(C) Mole percent enrichment of TCA intermediates from 500 µM [2,4-$^{13}C_2$]citrate in cancer cells grown in hypoxia for 48 hours (n=3).

(D) Palmitate mole percent enrichment from 500 µM [2,4-$^{13}C_2$]citrate in cancer cells grown in hypoxia for 48 hours (n=3).

(E) De novo synthesis of palmitate +/- 500 µM citrate in Huh7 (left) and HepG2 (right) cells grown in hypoxia over 48 hours (n=3).

Suc, succinate; Mal, malate; Asp, aspartate; α-KG, α-ketoglutarate. In (A-D) data are plotted as mean ± SD. Unless indicated, all data represent biological triplicates. Statistical significance is relative to normoxia (A,B) as determined by two-sided Student's t-test with *, P value < 0.05; **, P value < 0.01; ***, P value < 0.001, ****, P value < 0.0001. In (E) data are plotted as mean ± 95% confidence interval (CI). Statistical significance by non-overlapping confidence intervals, *. Data shown are from one of at least two separate experiments.

Huh7 *SLC13A5*-KO #1

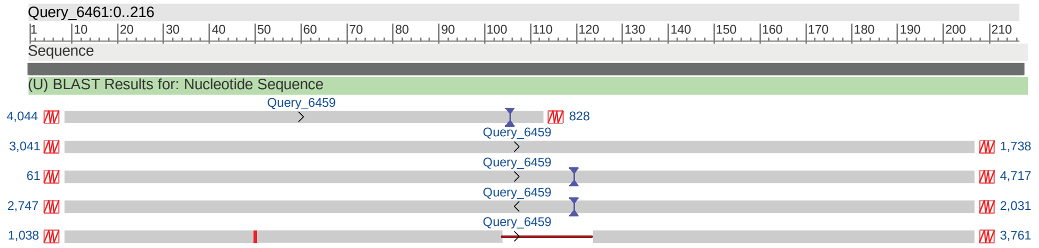

Huh7 *SLC13A5*-KO #2

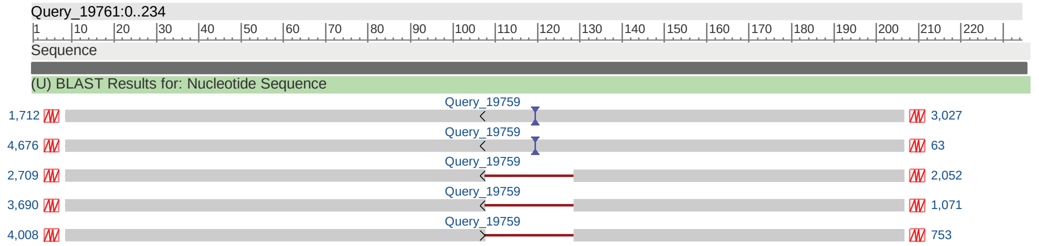

HepG2 *SLC13A5*-KO #1

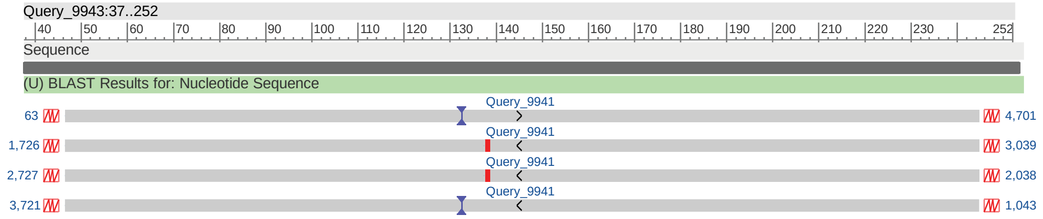

HepG2 *SLC13A5*-KO #2

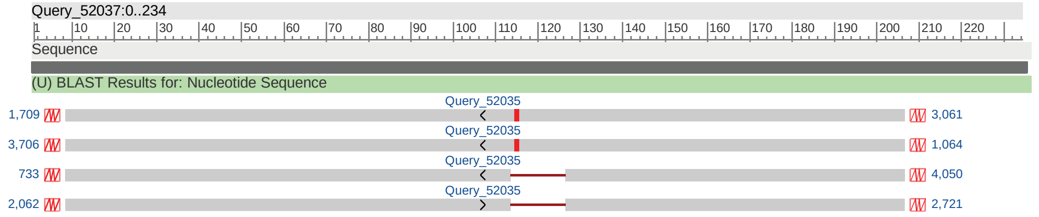

Supplemental Figure 3. Alignment of DNA sequences obtained from CRISPR/Cas9 HCC *SLC13A5*-KO clones. Related to Figure 5.

Sequences aligned using NCBI BLASTN suite (Agarwala et al., 2018). Results were visualized using NCBI Viewer 3.41.1.

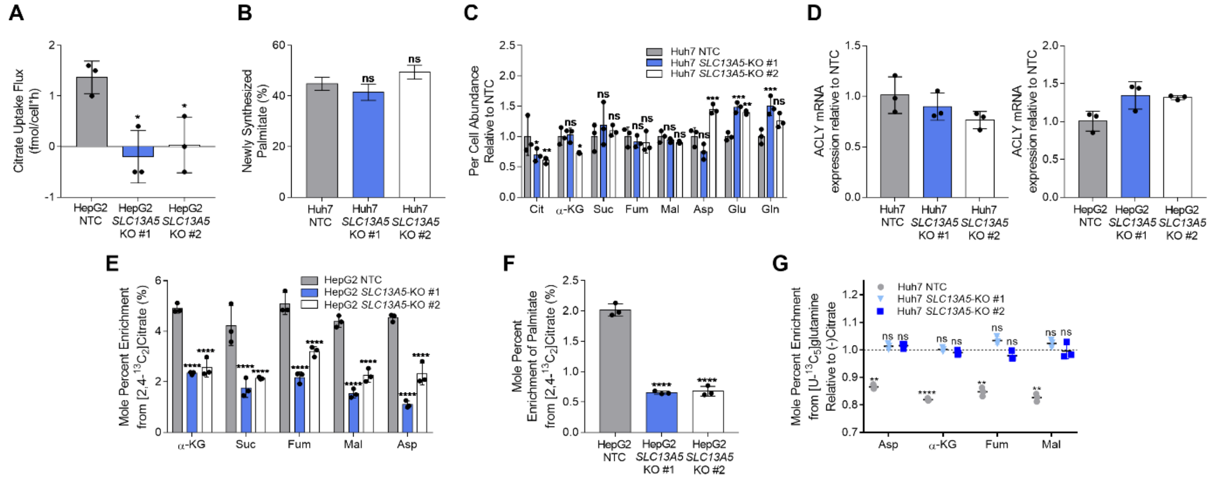

Supplemental Figure 4. NaCT supports extracellular citrate import and metabolism in hepatocellular carcinoma cells. Related to Figure 5.

(A) Citrate uptake flux in HepG2 NTC and *SLC13A5*-KO cells grown in hypoxia for 48 hours (n=3).

(B) De novo synthesis of palmitate in Huh7 NTC and *SLC13A5*-KO cells grown in hypoxia for 48 hours (n=3).

(C) Per cell abundance of metabolites in Huh7 NTC and *SLC13A5*-KO cells grown in hypoxia for 48 hours, relative to NTC (n=3).

(D) ACLY mRNA expression in NTC and *SLC13A5*-KO Huh7 (left) and HepG2 (right) cells grown in normoxia, relative to NTC (n=3).

(E) Mole percent enrichment of TCA intermediates from [2,4-$^{13}C_2$]citrate in HepG2 NTC and *SLC13A5*-KO cells grown in hypoxia for 48 hours (n=3).

(F) Mole percent enrichment of palmitate from [2,4-$^{13}C_2$]citrate in HepG2 NTC and *SLC13A5*-KO cells grown in hypoxia for 48 hours (n=3).

(G) Mole percent enrichment of TCA intermediates from [U-$^{13}C_5$]glutamine in Huh7 NTC and *SLC13A5*-KO cells +/- 500 µM citrate grown in hypoxia for 48 hours, relative to (-) citrate (n=3).

Cit, citrate; α-KG, α-ketoglutarate; Suc, succinate; Fum, fumarate; Mal, malate; Asp, aspartate; Glu, glutamate; Gln, glutamine. In (A,C-F) all graphs data are plotted as mean ± SD. Statistical significance is relative to NTC as determined by One-way ANOVA w/ Dunnet's method for multiple comparisons (A,C-F) or relative to (-) citrate as determined by two-sided Student's t-test (G) with *, P value < 0.05; **, P value < 0.01; ***, P value < 0.001, ****, P value < 0.0001. In (B) data are plotted as mean ± 95% confidence interval (CI). Statistical significance by non-overlapping confidence intervals, *. Unless indicated, all data represent biological triplicates. Data shown are from one of at least two separate experiments.

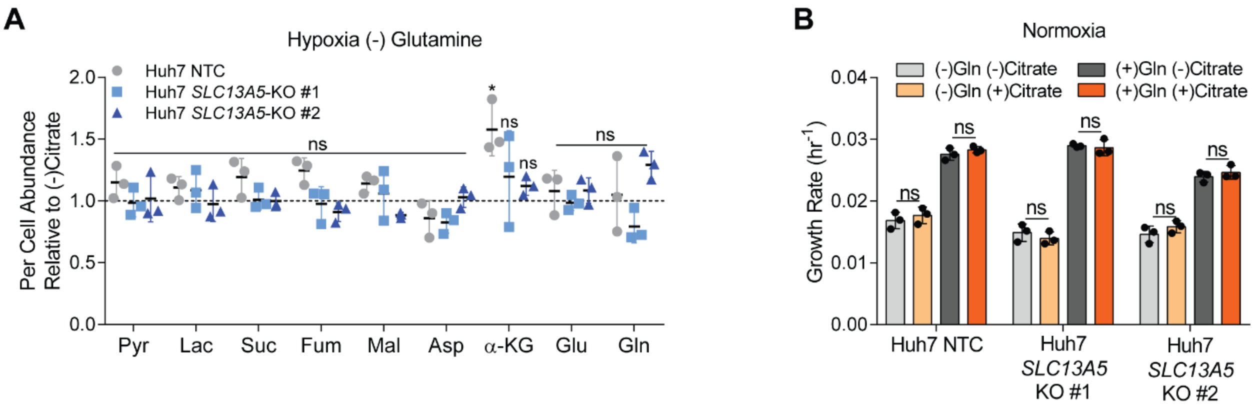

Supplemental Figure 5. NaCT facilitates growth under nutrient stress and resistance to zinc toxicity. Related to Figure 6.

(A) Per cell abundances of central carbon metabolites in Huh7 NTC and *SLC13A5*-KO cells grown without glutamine in hypoxia +/- 500 µM citrate for 48 hours, relative to (-) citrate (n=3).

(B) Growth rates of Huh7 NTC and *SLC13A5*-KO cells grown in high glucose DMEM +/- 4mM glutamine +/- 500 µM citrate in normoxia for 4 days (n=3).

Pyr, pyruvate; Lac, lactate; Suc, succinate; Fum, fumarate; Mal, malate; Asp, aspartate; α-KG, α-ketoglutarate; Glu, glutamate; Gln, glutamine. In all graphs data are plotted as mean ± SD. Statistical significance is determined by two-sided Student's t-test relative to (-) citrate (A); or determined by Two-way ANOVA w/ Tukey's method for multiple comparisons relative to (-) citrate (B) with *, P value < 0.05; **, P value < 0.01; ***, P value < 0.001, ****, P value < 0.0001. Unless indicated, all data represent biological triplicates. Data shown are from one of at least two separate experiments.

Table S1. Oligonucleotide sequences used in this study. Related to Figures 1 and 5.

Primer Name	Sequence	Application
ACLY (human) fwd	TCGGCCAAGGCAATTTCAGAG	qRT-PCR
ACLY (human) rev	CGAGCATACTTGAACCGATTCT	qRT-PCR
SLC13A5 (human) fwd	CTGCCACTCGTCATTCTGATG	qRT-PCR
SLC13A5 (human) rev	ATGTTGGTGTCCTTCATGTACTG	qRT-PCR
r18s (human) fwd	AGTCCCTGCCCTTTGTACACA	qRT-PCR
r18s (human) rev	CGATCCGAGGGCCTCACTA	qRT-PCR
Slc13a5 (rat) fwd	GGTGCACAGATGTCATCCCA	qRT-PCR
Slc13a5 (rat) rev	AGCATGTTGGTGTCCGTCAT	qRT-PCR
r18s (rat) fwd	AGAAACGGCTACCACATCCA	qRT-PCR
r18s (rat) rev	CTCGAAAGAGTCCTGTATTGT	qRT-PCR
SLC13A5 (human) PCR fwd	AGGCATCCCATAGTGACCCT	Target Site PCR Primer
SLC13A5 (human) PCR rev	CACAGAACTGCCGGAGTTGT	Target Site PCR Primer
sgRNA-NTC-fwd	GGCCGTGTTGCTGGATACGCC	CRISPR/Cas9
sgRNA-NTC-rev	GGCGTATCCAGCAACACGGCC	CRISPR/Cas9
sgRNA-SLC13A5-fwd	AGGCACAATGAATAGCAGGG	CRISPR/Cas9
sgRNA-SLC13A5-rev	CCCTGCTATTCATTGTGCCT	CRISPR/Cas9

Table S2. MFA of compartmental citrate catabolism in Huh7 hepatoma cells grown under hypoxia. Related to Figure 4.

Pathway/Reaction	Flux (fmol/cell/hr)	Lower bound (fmol/cell/hr)	Upper bound (fmol/cell/hr)
**Glycolysis (net fluxes)**			
Glc.x -> G6P	682	644.7	749.4
G6P -> F6P	682	264.1	719.3
F6P -> DHAP + GAP	681	593.1	714.9
DHAP -> GAP	681	593.1	714.9
GAP -> 3PG	1362	1245	1475
3PG -> PEP	1362	1245	1475
PEP -> Pyr.c	1362	1245	1475
Pyr.c -> Lac	1375	1306	1449
Lac -> Lac.x	1375	1306	1449
Pyr.c -> Ala	3.589	3.176	3.981
Pyr.m -> Ala	0.1301	0.05217	0.1871
**Pentose Phosphate Pathway (net fluxes)**			
G6P -> P5P + CO2	1.00E-07	0	430.2
P5P + P5P -> S7P + GAP	-0.4812	-0.5423	161.2
S7P + GAP -> F6P + E4P	-0.4812	-0.5423	161.2
P5P + E4P -> F6P + GAP	-0.4812	-0.5423	161.2
**Pyruvate Oxidation and Anaplerotic Reactions (net fluxes)**			
Cit.x -> Cit.e	9.648	9.099	10.42
Cit.e -> Cit.c	10.01	9.458	10.78
Pyr.c -> Pyr.m	4.631	4.097	5.264
Pyr.m + CO2 -> Oac.m	14.54	11.97	18.54
Mal.m -> Pyr.m + CO2	19.9	15.9	22.68
Mal.c -> Pyr.c + CO2	21.51	18.46	24.92
Pyr.m -> AcCoA.m + CO2	9.862	7.82	11.71
FAO -> AcCoA.m	7.791	5.094	9.553
Glu.m -> α-KG.m	-2.385	-10.69	45.04
Gln -> Glu.c	27.7	27.39	28.14
Gln.x -> Gln	29.69	29.53	29.86
Glu.c -> Glu.x	3.914	1.731	5.86
Glu.c -> Glu.m	-4.74E-11	-8.364	54.98
α-KG.c -> Glu.c	-21.4	-30.19	32.48
α-KG.m -> α-KG.c	-34.07	-42.24	-26.44
**TCA Cycle (net fluxes)**			
AcCoA.m + Oac.m -> Cit.m	17.65	15.99	19.19
Cit.m -> α-KG.m + CO2	-0.5944	-1.901	1.646
α-KG.m -> Suc + CO2	31.09	26.99	34.96

Suc -> Fum.m	31.09	26.99	34.96
Fum.m -> Mal.m	31.09	26.99	34.96
Mal.m -> Oac.m	5.665	-0.3408	9.143
Oac.m -> Asp.m	2.553	-1.954	4.303
Mal.c -> Oac.c	-2.83E-14	-17.13	4.285
Oac.c -> Asp.c	15.22	0.2183	17.8
Asp.c -> Fum.c	15.99	2.24E-13	18.6
Mal.c -> Fum.c	-15.99	-18.6	0
Mal.c -> Mal.m	-5.527	-14.49	-1.005
Asp.m -> Asp.c	2.553	-1.954	4.303
Cit.c -> α-KG.c + CO2	12.67	10.98	14.97
Cit.m -> Cit.c	18.25	16.38	20.73
**Biomass**			
Cit.c -> AcCoA.c + Oac.c	15.22	12.6	16.9
0*AcCoA.c + 0*AcCoA.c + 0*AcCoA.c + 0*AcCoA.c + 0*AcCoA.c + 0*AcCoA.c + 0*AcCoA.c + 0*AcCoA.c -> Palm.s	0.00769	0	Inf
Palm.d -> Palm.s	0.1254	0	Inf
114*Asp.c + 152*Glu.c + 152*Glu.m + 237*Ala + 127*Gln + 970*AcCoA.c + 92*P5P -> Biomass	0.01569	0.01299	0.01742
**Dilution/Mixing**			
0*Pyr.c -> Pyr.mnt	0.9488	0.9335	0.9643
0*Pyr.m -> Pyr.mnt	0.05123	0.03573	0.06649
0*Mal.c -> Mal.mnt	0.02303	1.00E-07	0.09788
0*Mal.m -> Mal.mnt	0.977	0.9021	1
0*Asp.c -> Asp.mnt	1	1.00E-07	1
0*Asp.m -> Asp.mnt	1.00E-07	0	1
0*Fum.m -> Fum.mnt	0.8298	0.6784	0.9363
0*Fum.c -> Fum.mnt	0.1702	0.0637	0.3216
0*Cit.m -> Cit.mnt	0.1439	1.00E-07	1
0*Cit.c -> Cit.mnt	0.8561	0	1
0*Glu.m -> Glu.mnt	1	0.9606	1
0*Glu.c -> Glu.mnt	1.00E-07	0	0.03941
0* α-KG.m -> α-KG.mnt	0.4998	1.00E-07	1
0* α-KG.c -> α-KG.mnt	0.5002	0	1
Pyr.mnt -> Pyr.fix	1	1	1
Asp.mnt -> Asp.fix	1	1	1
Mal.mnt -> Mal.fix	1	1	1
Fum.mnt -> Fum.fix	1	1	1
Cit.mnt -> Cit.fix	1	1	1
α-KG.mnt -> α-KG.fix	1	1	1

Glu.mnt -> Glu.fix	1	1	1
**Glycolysis (exchange fluxes)**			
G6P <- F6P	1.00E-07	0	Inf
DHAP <- GAP	0.00932	0	Inf
GAP <- 3PG	24.34	0	Inf
Pyr.c <- Lac	1.00E-07	0	Inf
**Pentose Phosphate Pathway (exchange fluxes)**			
P5P + P5P <- S7P + GAP	1.00E-07	0	Inf
S7P + GAP <- F6P + E4P	1.00E-07	0	Inf
P5P + E4P <- F6P + GAP	301.1	0	Inf
**Anaplerotic Reactions (exchange fluxes)**			
Cit.e <- Cit.c	0.3594	0.3217	0.382
Glu.m <- α-KG.m	1.563	0	Inf
Glu.c <- Glu.m	1.00E-07	0	61.6
α-KG.c <- Glu.c	1.00E-07	0	Inf
α-KG.m <- α-KG.c	157.6	0	Inf
**TCA Cycle (exchange fluxes)**			
Cit.m <- α-KG.m + CO2	1.00E-07	0	2.92
Suc <- Fum.m	0.001999	0	Inf
Fum.m <- Mal.m	3.35E+05	364.3	Inf
Mal.m <- Oac.m	69.2	32.19	178.2
Oac.m <- Asp.m	1.00E-07	0	Inf
Mal.c <- Oac.c	1.00E-07	0	Inf
Oac.c <- Asp.c	0.05184	0	Inf
Mal.c <- Fum.c	1768	0	Inf
Mal.c <- Mal.m	1.00E-07	0	4.497
Asp.m <- Asp.c	0.3676	0	Inf
Cit.c <- α-KG.c + CO2	1.00E-07	0	2.124
Cit.m <- Cit.c	1.00E-07	0	Inf

SSR = 170.4
Expected SSR = [141.2 214.6] (95.0% conf., 176 DOF)

Compartment abbreviations: **c**, cytosol; **m**, mitochondrial; **mnt**, measured (utilized to indicate that measured MIDs reflect aggregated metabolite labeling across all compartments); **x**, extracellular; **e**, extracellular exchange intermediate (utilized in order to allow for incorporation of both citrate uptake and efflux).

Table S3. Metabolite fragments considered in MFA. Related to Figure 4.

Metabolite	Carbons	Formula	m/z
Pyruvate	1,2,3	$C_6H_{12}O_3NSi$	174
Lactate	2,3	$C_{10}H_{25}O_2Si_2$	233
Lactate	1,2,3	$C_{11}H_{25}O_3Si_2$	261
Alanine	2,3	$C_{10}H_{26}ONSi_2$	232
Alanine	1,2,3	$C_{11}H_{26}O_2NSi_2$	260
α-KG	1,2,3,4,5	$C_{14}H_{28}O_5NSi_2$	346
Malate	1,2,3,4	$C_{18}H_{39}O_5Si_3$	419
Succinate	1,2,3,4	$C_{12}H_{25}O_4Si_2$	289
Fumarate	1,2,3,4	$C_{12}H_{23}O_4Si_2$	287
Aspartate	1,2	$C_{14}H_{32}O_2NSi_2$	302
Glutamate	1,2,3,4,5	$C_{19}H_{42}O_4NSi_3$	432
Glutamine	1,2,3,4,5	$C_{19}H_{43}O_3N_2Si_3$	431
Palmitate	1-16	$C_{17}H_{34}O_2$	270
